# Impact of dairy fat manipulation on endothelial function and lipid regulation in human aortic endothelial cells exposed to human plasma samples: an in vitro investigation from the RESET study

**DOI:** 10.1007/s00394-023-03284-9

**Published:** 2023-12-13

**Authors:** Oonagh Markey, Alba Garcimartín, Dafni Vasilopoulou, Kirsty E. Kliem, Colette C. Fagan, David J. Humphries, Susan Todd, David I. Givens, Julie A. Lovegrove, Kim G. Jackson

**Affiliations:** 1 Hugh Sinclair Unit of Human Nutrition and Institute for Cardiovascular and Metabolic Research, University of Reading, Reading, RG6 6DZ UK; 2 Department of Animal Sciences, University of Reading, Reading, RG6 6AR UK; 3 Institute for Food, Nutrition, and Health, University of Reading, Reading, RG6 6AR UK; 4 Department of Mathematics and Statistics, University of Reading, Reading, RG6 6AX UK; 5 Present Address: School of Sport, Exercise, and Health Sciences, Loughborough University, Loughborough, LE11 3TU UK; 6 Present Address: Pharmacology, Pharmacognosy and Botany Department, Pharmacy School, Complutense University of Madrid, 28040 Madrid, Spain

**Keywords:** Cell adhesion molecules, Dairy fatty acid manipulation, Human aortic endothelial cells, Nitric oxide, Real-time PCR

## Abstract

**Purpose:**

Longer-term intake of fatty acid (FA)-modified dairy products (SFA-reduced, MUFA-enriched) was reported to attenuate postprandial endothelial function in humans, relative to conventional (control) dairy. Thus, we performed an in vitro study in human aortic endothelial cells (HAEC) to investigate mechanisms underlying the effects observed in vivo.

**Methods:**

This sub-study was conducted within the framework of the RESET study, a 12-week randomised controlled crossover trial with FA-modified and control dairy diets. HAEC were incubated for 24 h with post-intervention plasma samples from eleven adults (age: 57.5 ± 6.0 years; BMI: 25.7 ± 2.7 kg/m^2^) at moderate cardiovascular disease risk following representative sequential mixed meals. Markers of endothelial function and lipid regulation were assessed.

**Results:**

Relative to control, HAEC incubation with plasma following the FA-modified treatment increased postprandial NOx production (*P*-interaction = 0.019), yet up-regulated relative E-selectin mRNA gene expression (*P*-interaction = 0.011). There was no impact on other genes measured.

**Conclusion:**

Incubation of HAEC with human plasma collected after longer-term dairy fat manipulation had a beneficial impact on postprandial NOx production. Further ex vivo research is needed to understand the impact of partial replacement of SFA with unsaturated fatty acids in dairy foods on pathways involved in endothelial function.

**Supplementary Information:**

The online version contains supplementary material available at 10.1007/s00394-023-03284-9.

## Introduction

Despite their relatively high SFA content, evidence from longitudinal observational studies indicates that increased consumption of whole-fat dairy products, except butter, are associated with a neutral or reduced risk of cardiovascular diseases (CVD) [1–3]. This finding may be partly explained by the complex dairy food matrix, which is comprised of components including micronutrients, proteins, bioactive peptides, and phospholipids [1, 2, 4].

Supplementation of the dairy cow diet with plant oil or oilseeds is a reformulation initiative for partial replacement of milk SFAs with unsaturated fatty acids (FAs), primarily in the form of *cis*-MUFA [1, 5, 6]. In the REplacement of SaturatEd fat in dairy on Total cholesterol (RESET) study, we demonstrated that longer-term (12-wk) consumption of SFA-reduced, MUFA-enriched (FA-modified) milk, cheese and butter (∼ 41 g/day dairy fat) attenuated the rise in fasting serum LDL-cholesterol concentrations, enhanced endothelial-dependent flow-mediated dilatation (FMD) and plasma nitrite concentrations among a cohort of adults at moderate CVD risk, relative to conventional dairy products [1]. More recently, we presented evidence from the same ‘at risk’ cohort which indicated that longer-term intake of FA-modified dairy products, enhanced the postprandial (non-fasted) circulating apolipoprotein (apo)B response to representative sequential meals, but attenuated the %FMD response observed with the conventional dairy treatment [5]. In the context of dietary SFA replacement and cardiometabolic disease risk, this highlights the importance of considering biomarker assessment in the postprandial state [5].

To determine mechanisms underlying the impact of dietary FAs on endothelial function, in vitro studies have been performed using human aortic or umbilical vein endothelial cells. Livingstone et al. [7] conducted the first in vitro study to examine the effect of concentrations of individual dairy FA and FA mixtures extracted from conventional and FA-modified cheese on biomarkers of endothelial function in human aortic endothelial cells (HAEC). Building on this previous work [7] and to complement the findings from our human study [1, 5], we exposed HAEC to ex vivo plasma to provide more physiologically relevant insights into the mechanisms underlying the impact of FA-reformulated dairy foods on vascular function, nitric oxide (NO) production (a potent vasodilator) and CVD development. Within the framework of the RESET study, the aim of this in vitro work was to investigate the longer-term impact of consuming FA-modified compared to conventional dairy food treatments on markers of endothelial function and expression of genes involved in lipid regulation and endothelial activation in HAEC incubated for 24 h with plasma samples isolated from adults with moderate CVD risk after consumption of representative sequential high-fat meals.

## Materials and methods

### Participants and study samples

Details of the RESET double-blind, cross-over, acute-within-chronic study (NCT02089035), including the eligibility criteria and the study protocol, have been detailed elsewhere [1, 5, 8]. This study was given a favourable ethical opinion for conduct by the University of Reading Research Ethics Committee (Ref No.: 13/43) and was performed in accordance with the ethical standards laid down in the 1964 Declaration of Helsinki and its later amendments.

Informed consent was obtained from all participants included in the study. Briefly, males and females (aged 25–70 years; BMI 19–32 kg/m^2^) with a moderate CVD risk (≥ 50% above the population mean), as assessed using a modified Framingham risk score, were randomly assigned to consume the FA-modified or conventional (control) dairy products (milk, cheese, and butter; ∼ 41 g/day of dairy fat) for 12 weeks, with an 8-week washout period between interventions. The details of our high-oleic sunflower oil dairy cow feeding strategy, as well as the nutrient profile of the FA-modified and control dairy products are reported elsewhere [1, 6, 9]. The high-fat, high-dairy dietary exchange was isoenergetic [38% of total energy intake (%TE) from total fat] but varied in FA composition (FA-modified dairy diet: 16%TE SFA; 14%TE MUFA; control dairy diet: 19%TE SFA; 11%TE MUFA). The dietary exchange was achieved by asking participants to replace their habitual dairy foods, cooking oil/spreads, and snacks with the study dairy products, specifically 340 g/day of ultra-high temperature (UHT) milk, 45 g/day of Cheddar cheese, and 25.1 g/day (FA-modified diet) or 21.5 g/day (control diet) of butter [1, 9]. A 480-min postprandial study visit was conducted at the beginning and end of each 12-week intervention period, with a sequential two-meal dairy fat challenge representative of the assigned dietary interventions consumed by participants at 0 min (breakfast) and 330 min (lunch), as described previously [5, 8]. The energy and nutrient composition of the sequential high-fat, high-dairy mixed-meal challenges are presented in Table 1. The standardised breakfast meal consisted of a toasted sandwich prepared with white bread (75 g; Kingsmill; Allied Bakeries, UK), Cheddar cheese (32.6 g) and butter (FA-modified: 32.6 g; control: 29.4 g)], cornflakes (38 g, Kellogg's UK) served with UHT milk (195 g), and a strawberry milkshake [prepared with UHT milk (330 g) and strawberry sauce (19 g; Askeys; Silver Spoon Company, UK)]. The standardised lunch meal consisted of a toasted sandwich [white bread (60 g; Kingsmill; Allied Bakeries, UK), Cheddar cheese (15 g) and butter (FA-modified: 19.8 g; control: 18.6 g)] and a strawberry milkshake [UHT milk (FA-modified: 352 g; control: 350 g) and strawberry sauce (27 g; Askeys; Silver Spoon Company, UK)].

**Table 1:** Energy and nutritional composition of the sequential high-fat mixed-meal challenges (breakfast at 0 min and lunch at 330 min) that incorporated the FA-modified and conventional (control) dairy products

	FA-modified			Control		
	Breakfast	Lunch	Total	Breakfast	Lunch	Total
Energy^a^, MJ	4.3	2.6	6.9	4.1	2.5	6.6
Total fat^a^, g	50.6	30.6	81.2	49.9	30.3	80.2
SFA^b^, g	24.5	14.8	39.3	31.7	19.1	50.8
MUFA^b^, g	20.0	12.1	32.1	12.3	7.4	19.7
TFA^b^, g	3.9	2.6	6.5	2.2	1.4	3.6
PUFA^b^, g	2.9	1.8	4.7	2.8	1.8	4.6
Carbohydrate^a^, g	105.9	64.6	170.5	101.4	63.3	164.7
Free sugars, g	16.5	22.3	38.8	15.0	21.5	36.5
Protein^a^, g	36.1	20.9	57.0	39.7	19.6	59.3

Values represent total energy and macronutrient quantities of each test meal according to FA-modified and control dairy diet. Adapted from Markey et al. [8]. MUFA, monounsaturated fatty acids; PUFA, polyunsaturated fatty acids; SFA, saturated fatty acids; TFA, *trans* fatty acids

^a^Energy, total fat, carbohydrate, and protein content of dairy product samples were assessed in duplicate by SGS UK Ltd. (Ealing, London; ISO 17025 accredited laboratory)

^b^Lipid extracted from dairy products were analysed in triplicate for FA composition by GC-flame ionisation detection, as previously reported [6]

For this in vitro investigation, blood samples were collected into lithium heparin tubes at − 30, 180, 300 and 420 min during the postprandial study visits conducted at the end of the 12-wk FA-modified and control dairy intervention periods only (i.e., post-intervention: week 12 and week 32) (Supplemental Fig. 1). These study visits and samples were chosen since they reflected the longer-term effects of the dairy fat interventions on the postprandial outcome measures and coincided with the timing of the FMD measurement and collection of blood samples for markers of endothelial function [5].

The blood tubes were centrifuged at 1750×*g* (3000 rpm). Subsequently, plasma was aliquoted and stored at − 80 °C prior to incubation with cell cultures. Plasma samples were chosen from a subset of participants from Cohort 1 of the RESET study (*n* = 11, 6 males and 5 females) according to the sequence of the dietary intervention allocation (*n* = 6 randomized to control dairy diet or *n* = 5 to modified dairy diet during their first study period) and matched according to age and BMI. To align our in vitro study findings to physiological responses observed in our human study, in vivo data presented within this report are from the same RESET participant subgroup who were selected for the in vitro cell study. Subgroup samples following 12-week exposure to FA-modified and conventional (control) dairy diets (− 30, 180-, 300- and 420-min timepoints) were selected. We focused on outcomes that were linked to endothelial function and lipid regulation, including %FMD response and serum/plasma lipids (triacylglycerol (TG), apoB, non-esterified fatty acid (NEFA)), glucose, insulin, markers of NO availability (nitrite and nitrate), markers of endothelial activation (soluble vascular cell adhesion molecule (VCAM)-1, soluble intercellular adhesion molecule-1 (ICAM)-1, E-selectin, P-selectin) and total lipid FA profile, as described in detail elsewhere [1, 5].

### Endothelial cell culture

Clonetics™ single donor HAEC (Lot no. 0000370752, Lonza Biologics Plc, Slough, UK) were cultured in complete medium: endothelial basal medium™-2 with endothelial growth medium™-2 SingleQuots™ Supplements [2% (v/v) fetal bovine serum (FBS), hydrocortisone, human fibroblast growth factor-B, vascular endothelial growth factor, insulin-like growth factor-1, ascorbic acid, human epidermal growth factor, gentamicin sulphate amphotericin B-1000 and heparin].

For experiments, only endothelial cells passaged less than four times were used. HAEC were seeded into 12 well plates (for the measurement of NO and E-selectin in the cell culture supernatant) or 6 well plates (for the determination of gene expression) at ~ 5000 cells/cm^2^ and grown to 80% confluence in a complete medium. Cells were then washed with HEPES buffered saline solution and maintained in serum-free medium for 3 h prior to incubation for 24 h with a complete medium containing 2% of lithium heparinized plasma. During the development of our in vitro studies, we tested different concentrations of fasting and postprandial serum and lithium heparinized plasma (2, 5 and 10%) on cell viability and endothelial NO production. Although there was a concentration effect of plasma on NO production, this was not evident with serum. Furthermore, serum and higher concentrations of plasma (5 and 10%) were found to interfere with the NO measurement. Therefore, a final concentration of 2% lithium heparinized plasma in the cell culture medium was chosen which agrees with previous in vitro studies [10, 11]. All experiments were performed using duplicate wells. After the 24 h incubation, the 12 well plates were put on ice and the cell culture supernatant was collected, aliquoted and stored at − 80 °C until the measurement of nitrate, nitrite, and E-selectin. The cells were then washed with ice cold HBSS before incubation with 50 µl/well of 1 × lysis buffer (20 mM Tris–HCl (pH 7.5), 150 mM NaCl, 1 mM Na_2_EDTA, 1 mM EGTA, 1% Triton, 2.5 mM sodium pyrophosphate, 1 mM β-glycerophosphate, 1 mM Na_3_VO_4_ and 1 µg/ml leupeptin) (New England Biolabs, Hitchin, UK) supplemented with 1 mM PMSF for 5 min. The cells were scraped, transferred to Eppendorf tubes, and sonicated twice for 30 s before centrifugation at 14,000×*g* (12,100 rpm) for 10 min at 4 °C. The supernatant was isolated and stored at − 80 °C until the protein content of the cell lysate was determined using the Pierce™ BCA protein assay kit (ThermoFisher Scientific). Nitrate and nitrite concentrations were measured in the cell culture supernatants by ozone-based chemiluminescence (model 88 AM, Eco Physics) as previously described [12, 13]. The nitrate and nitrite concentrations were then summed to calculate total nitric oxide (NOx), as a biomarker of NO production. E-selectin was measured using a Quantikine ELISA (R&D Systems Europe Ltd) according to the manufacturer’s instructions for cell culture supernatants. Both NOx and E-selectin concentrations were corrected for the total protein concentration in each well.

For the 6 well plates, the cells were washed with HBSS before the addition of 300 µl of trypsin/EDTA solution (0.025%) per well and incubated at 37 °C for 5 min. HBSS (300 µl) was then added, the plates scraped, and cells transferred to Eppendorf tubes before storage at − 80 °C. For the isolation of total RNA, the cells were defrosted on ice and centrifuged at 16,100×*g* (13,200 rpm) for 5 min at 4 °C. The supernatant was carefully removed, and the RNA was extracted from the cells using the Qiagen RNeasy Mini Kit and QIA shredder (Qiagen Ltd., Crawley, UK) using protocols recommended by the manufacturer. cDNA was generated from 0.4 to 1.1 μg samples of total RNA at 42 °C for 50 min (reaction volume 20 μl) using oligo dT_12–18_ primer (Invitrogen Ltd, Paisley, UK) and reverse transcriptase (Superscript II; Invitrogen Ltd) using protocols recommended by the manufacturer.

Real time-PCR was performed, as previously described [14–17]. The primer sequences for β-actin, VCAM-1 and E-selectin were obtained from Shaw et al. [16], endothelial nitric oxide synthase (eNOS) from Miyomoto et al. [18], ICAM-1 from Virgili et al. [19], insulin receptor from Livingstone et al. [7], LDL-receptor (LDL-R) from Jackson et al. [20], and sirtuin 1(SIRT1) from Jung et al. [21]. The mRNA expression of each target gene was normalized to the β-actin expression and the data represent the postprandial fold change in mRNA expression relative to the baseline (fasting sample) following each 12-week dairy intervention, which is arbitrarily defined as 1.

### Statistical analyses

Data are presented as unadjusted means ± SEMs unless otherwise stated. Variables were assessed for normality, and log transformed where necessary. Statistical analyses were performed using SAS On Demand for Academics (SAS Institute, Inc.) and IBM SPSS Statistics 28.0 (Statistical Product and Service Solutions; IBM Corp.).

Data were analyzed using linear mixed models (PROC MIXED). Treatment x time interaction effects were first included in the model and retained where found to be significant. In the absence of a significant interaction, the interaction term was removed from the model so that the overall treatment effect could be evaluated. Fixed—(period, time, treatment, sex, age, and BMI) and random—(participant) effect covariates were retained in all linear mixed models, regardless of their degrees of significance. Results were deemed significant at *P* < 0.05.

For outcomes where differences in baseline (fasting) values were observed between study visits i.e., plasma nitrite, and total lipid FA (14:0, 16:0, 18:0, *trans*-MUFAs, *trans*-9 18:1, *trans*-10 18:1 and *trans*-11 18:1), incremental postprandial treatment × time interactions/overall treatment effects were assessed by subtracting fasting (baseline) from postprandial values (i.e., the baseline value was treated as ‘0’) [22].

## Results

### Human study

Baseline (fasting) characteristics of eleven participants (age: 57.5 ± 6.0 years; BMI: 25.7 ± 2.7 kg/m^2^) following each intervention period are presented in Supplemental Table 1. Serum total cholesterol concentrations were lower, with a tendency for a similar reduction in LDL-C concentration, following the FA-modified diet, relative to the control diet (*P* = 0.037 and *P* = 0.068, respectively). In line with findings from our full RESET study cohort [5], fasting plasma nitrite concentrations were higher following the FA-modified, relative to the control diet (*P* = 0.01). There were no other significant differences in characteristics and fasting serum biomarkers at the beginning of each post-intervention acute study visit.

The postprandial TG time-course response was significantly lower after consumption of the FA-modified, relative to the control dairy treatment (12-week diet and representative test meals; *P*-treatment < 0.0001; Supplemental Fig. 2A). There was no differential impact of the two dairy treatments on time-course profiles of postprandial plasma apoB, NEFA, glucose or insulin responses (data not shown). The postprandial and incremental postprandial nitrite time-course response were significantly lower after the FA-modified, relative to the control dairy treatment (*P*-interaction = 0.014 and 0.015 (Supplemental Fig. 2B, respectively). There was no effect of dairy treatment on %FMD response, plasma nitrate or markers of endothelial activation (data not shown).

Differential effects were evident for the postprandial plasma total lipid FA profile time-course responses following the two dairy treatments (12-week diet and representative test meals) (see Supplemental Figs. 3, 4). The postprandial and incremental postprandial proportion of 16:0 [palmitic acid (PA)] was significantly lower after the FA-modified when compared to the control dairy treatment (Incremental *P*-treatment < 0.0001, respectively). The postprandial proportion of total *cis*-MUFA and *cis*-9 18:1 (oleic acid (OA)) was significantly higher after the FA-modified relative to the control dairy treatment (*P*-treatment = 0.012 and 0.032, respectively). The postprandial and incremental postprandial proportion of *trans*-MUFA and *trans*-10 18:1 (octadecenoic acid) response was significantly higher after the FA-modified relative to the control dairy treatment (Incremental *P*-interaction: 0.029 and 0.007, respectively). Relative to the control, the postprandial proportion of 18:0 (stearic acid) and *trans*-9 18:1 (elaidic acid) was significantly higher after the FA-modified dairy treatment, with significance lost or only a tendency for significance observed for the incremental postprandial responses (Incremental *P*-treatment = 0.868 and 0.055, respectively). Relative to the FA-modified dairy treatment, the proportion of *trans*-11 18:1 (vaccenic acid) in the postprandial plasma lipid pool was higher after the control dairy treatment (*P*-treatment = 0.006, but significance was lost for the incremental postprandial response (*P* > 0.05).

### Cell study

**Fig. 1:**
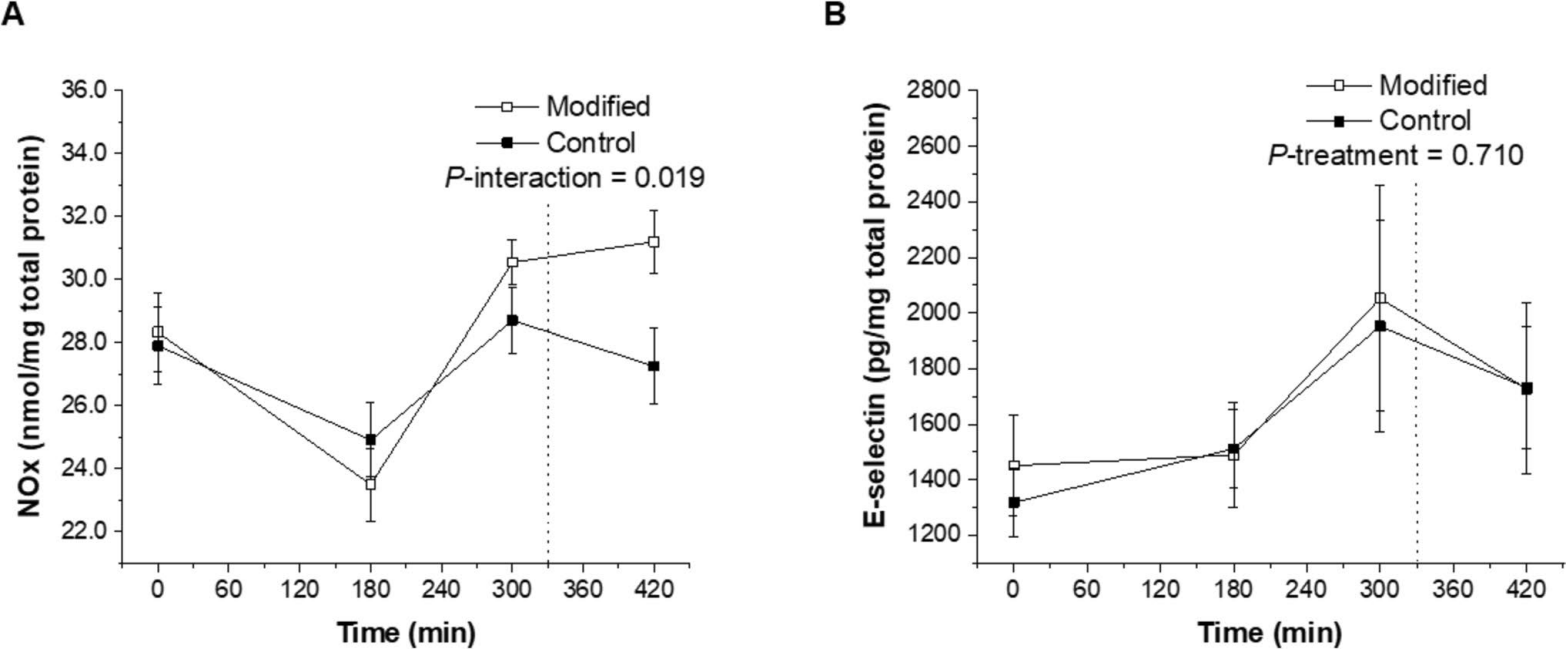
Postprandial NOx (**A**) and E-selectin (**B**) concentrations measured in the cell culture supernatant following 24-h incubation of human aortic endothelial cells with plasma samples isolated from a subset of RESET participants who were exposed to sequential high-fat mixed-meal challenges (breakfast at 0 min and lunch at 330 min) representative of the FA-modified and conventional (control) dairy diets following each 12-week dietary intervention. All experiments were performed using duplicate wells and concentrations were corrected for the protein content in each well. Values are untransformed and unadjusted means ± SEMs (*n* = 11). The dotted lines represent the timing of the second meal (330 min). Linear mixed-model analysis was used to explore the effects of treatment and time, with an adjustment made in all cases for fixed—(period, time, treatment, sex, age, and BMI) and random—(participant) effect covariates. *P* < 0.05 was deemed significant. *FA* fatty acid, *NOx* total nitric oxide

Exposure of HAEC to postprandial plasma samples collected after the FA-modified treatment (diet and representative test meals) promoted a significantly higher NOx concentration in the cell culture supernatant than those after the control dairy treatment (*P*-interaction = 0.019; Fig. 1A). There was no differential effect of dairy FA composition on cell culture supernatant E-selectin concentrations following exposure to postprandial plasma samples (*P* > 0.05; Fig. 1B).

**Fig. 2:**
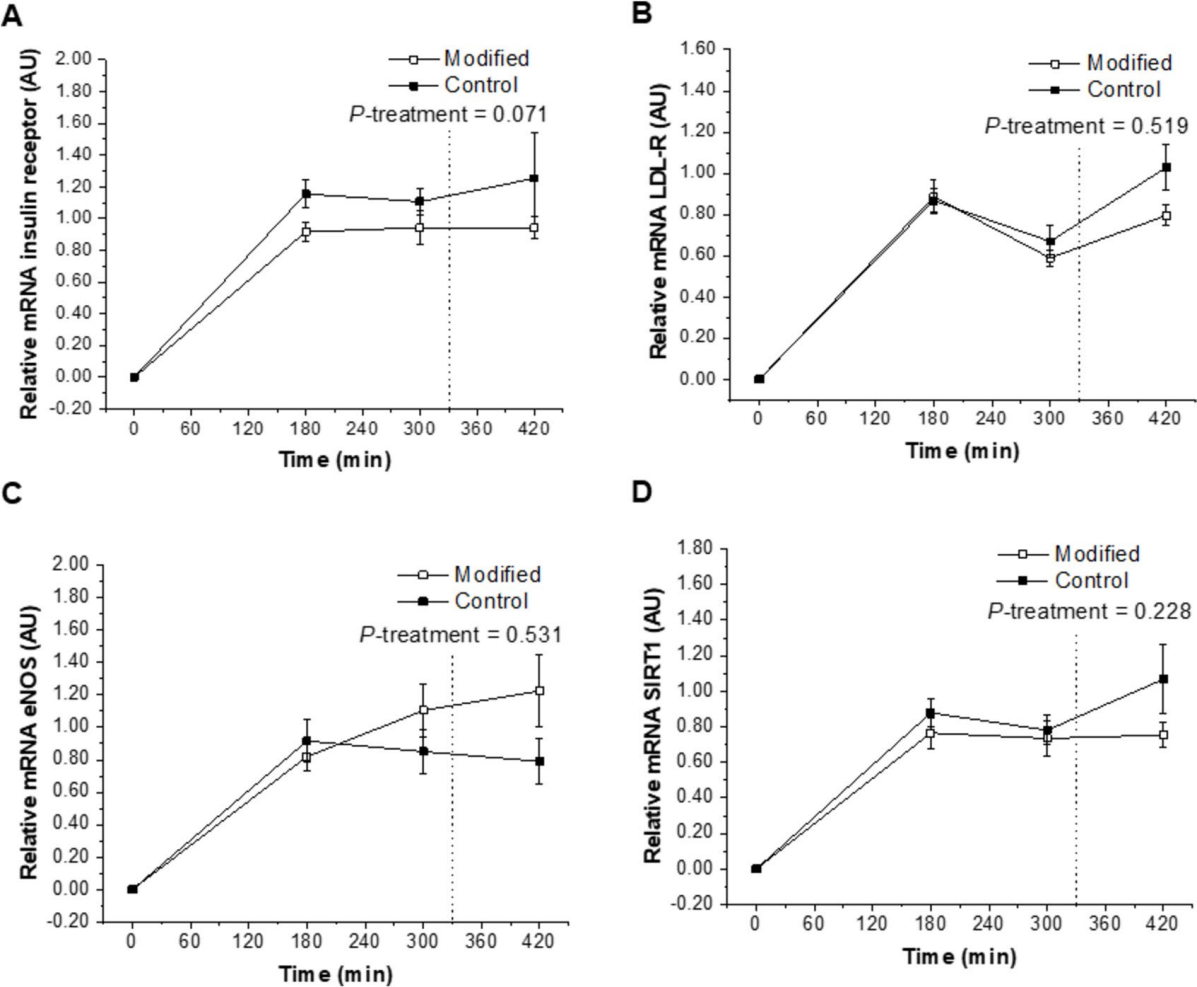
Relative postprandial mRNA expression of insulin receptor (**A**), LDL-R (**B**), eNOS (**C**) and SIRT1 (**D**) in response to 24-h incubation of human aortic endothelial cells with plasma samples isolated from a subset of participants who were exposed to sequential high-fat mixed-meal challenges (breakfast at 0 min and lunch at 330 min) representative of the FA-modified and conventional (control) dairy diets following each 12-week dietary intervention. The real-time PCR was performed in duplicate. Values are untransformed and unadjusted means ± SEMs normalized for beta-actin and then calculated relative to the fasting timepoint on each visit (*n* = 11), which are arbitrarily set as 1. Linear mixed-model analysis was used to explore the effects of treatment and time, with an adjustment made in all cases for fixed—(period, time, treatment, sex, age, and BMI) and random—(participant) effect covariates. *P* < 0.05 was deemed significant. *eNOS* endothelial nitric oxide synthase, *FA* fatty acid, *LDL-R* LDL-receptor, *SIRT1* sirtuin 1

**Fig. 3:**
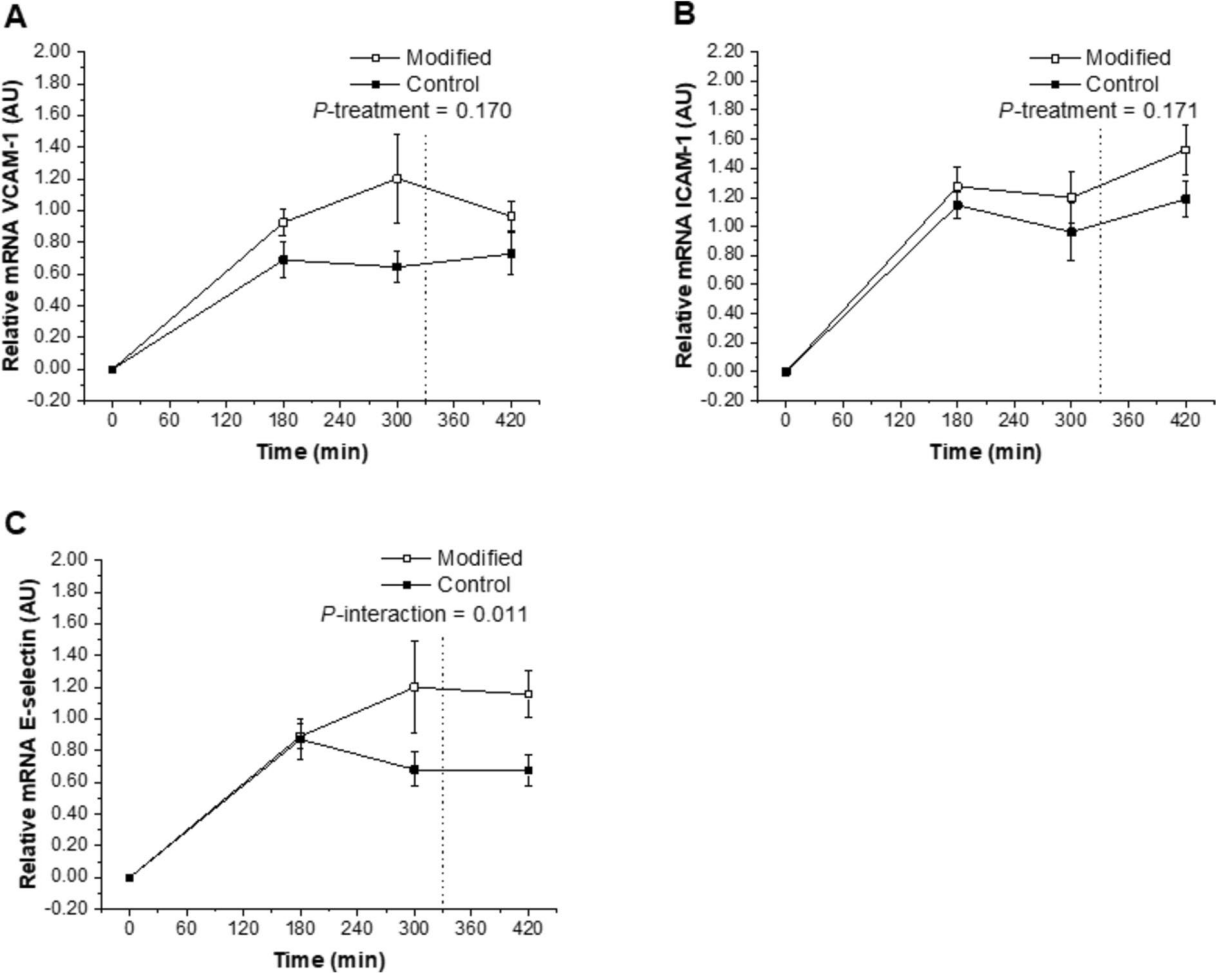
Relative postprandial mRNA expression of VCAM-1 (**A**), ICAM-1 (**B**) and E-selectin (**C**) in response to 24-h incubation of human aortic endothelial cells with plasma samples isolated from a subset of participants who were exposed to sequential high-fat mixed-meal challenges (breakfast at 0 min and lunch at 330 min) representative of the FA-modified and conventional (control) dairy diets following each 12-week dietary intervention. The real-time PCR was performed in duplicate. Values are untransformed and unadjusted means ± SEMs normalized for beta-actin and then calculated relative to the fasting timepoint on each visit (*n* = 11), which are arbitrarily set as 1. Linear mixed-model analysis was used to explore the effects of treatment and time, with an adjustment made in all cases for fixed—(period, time, treatment, sex, age, and BMI) and random—(participant) effect covariates. *P* < 0.05 was deemed significant. *eNOS* endothelial nitric oxide synthase, *FA* fatty acid, *ICAM-1* intercellular adhesion molecule-1, *VCAM-1* vascular cell adhesion molecule-1

Exposure of HAEC to postprandial plasma collected after the FA-modified dairy treatment (diet and representative test meals) was shown to up-regulate the relative mRNA expression of E-selectin compared with the control dairy treatment (*P*-interaction = 0.011; Fig. 3C). The FA composition of the dairy treatment had no effect on relative mRNA expression of the insulin receptor, LDL-R, eNOS, SIRT1, VCAM-1, and ICAM-1 after incubation with the postprandial plasma samples (*P* > 0.05; Figs. 2A–D, 3A, B).

## Discussion

This study is the first to compare the impact of exposing HAEC to plasma from participants at moderate CVD risk after chronic intake of FA-modified, relative to a conventional dairy treatment (12-week diet and representative test meals) on markers of endothelial function and gene expression of targets involved in lipid regulation and endothelial activation. Incubation with plasma collected following the SFA-reduced, MUFA-enriched FA-modified dairy treatment increased NOx concentrations in the cell culture supernatant when compared to plasma samples collected after intake of conventional dairy foods. This observation suggests that FA-modified dairy products had a favourable impact on endothelial function in HAEC, which was influenced in part by increased NO bioavailability [23, 24]. We previously observed that exposure of HAEC to OA promoted signaling by the PI3K/Akt pathway to increase eNOS phosphorylation at Ser^1177^, relative to PA, linoleic acid, and stearic acid [25]. It is possible that the higher postprandial proportion of plasma total lipid OA that HAEC were exposed to following our MUFA-enriched, SFA-reduced dairy treatment acted in a similar manner to OA in the above-mentioned study [25]. Conversely, in the sub-group of participants chosen for the in vitro study, we observed the postprandial plasma nitrite response to be significantly lower after the FA-modified, relative to the control dairy treatment. In our full human study cohort [5], we previously reported that the FA-modified treatment attenuated the postprandial %FMD response and tended to decrease the plasma nitrite response, relative to conventional dairy. Discrepancies between human and in vitro study findings could be partly explained by the primary endothelial cell models lacking the underlying smooth muscle cells from the vascular lumen and not fully representing the vascular endothelium and associated in vivo pathophysiological mechanisms [25]. This acknowledges that many factors can impact eNOS gene expression in vivo that could not be replicated in vivo such as shear stress and altered patterns of mechanical stretching of the vasculature (cyclic stretching) which can lead to changes in vascular tone and gene regulation [26]. Inconsistencies could also be due to the cell work measuring NOx, whereas the human study measured nitrate and nitrite separately. Our study is unique as we have exposed endothelial cells to human plasma samples following longer-term consumption of whole dairy foods. While we acknowledge that findings may not be directly comparable to studies that have exposed cells to single FA or FA mixtures, the current study does extend on a previous in vitro study which reported that 5-h incubation of HUVEC with PA (the predominant FA in conventional dairy products) dose-dependently inhibited NO release [27]. Additionally, individual FA affected NO concentrations in HAEC cell supernatant, with an increase following 24-h incubation with PA when compared to other FA treatments [7]. It is known that endothelial cells are amenable to alterations in the FA composition of plasma lipid fractions [27, 28]. Our human data indicated that, relative to conventional dairy, the postprandial proportion of PA in the total plasma lipid FA pool was significantly lower, and OA was higher, following the FA-modified dairy treatment. Although not measured directly in our cell experiment, it could be speculated that our dairy treatments affected FA incorporation into endothelial cells. Indeed, based on their observations in a HUVEC experiment, Carluccio et al. [29] concluded that exposure to OA may partly contribute to the prevention of atherogenesis through displacement and replacement of SFA in endothelial cell membranes.

We found that exposure of HAEC to postprandial plasma collected after the FA-modified dairy treatment up-regulated relative gene expression of the inflammatory marker E-selectin, but not E-selectin production, when compared to the control dairy treatment. Livingstone et al. reported that physiological concentrations of dairy FA mixtures affected E-selectin production (but not gene expression), with lower concentrations observed after incubation of HAEC with lipid extracted from conventional vs. FA-modified cheese [7]. The deleterious effects of exposure to PA, but not OA, have been reported by showing increased E-selectin mRNA and E-selectin cell surface protein expression in HUVEC and HAEC, respectively [16, 30]. Although our findings are not in agreement with in vitro studies which demonstrated that human endothelial cell treatment with OA had neutral effects or dampened pro-inflammatory responses [16, 30, 31], we have previously shown that longer-term intake of FA-modified dairy foods attenuated the postprandial %FMD response observed with the conventional dairy treatment, without affecting circulating postprandial adhesion molecules, including E-selectin [5]. Our finding may be partly linked to the TFA content and a shift in the *trans*-isomer profile of the FA-modified dairy products [5], which led to a greater postprandial proportion of plasma *trans-*MUFA and *trans*-10 18:1 (octadecenoic acid) and a tendency towards greater *trans*-9 18:1 (elaidic acid) proportion (particularly at the later postprandial timepoints), relative to the control dairy treatment. It is recognized that increased consumption of TFA, specifically elaidic acid, the major industrial *trans*-fat found in partially hydrogenated vegetable oils, is associated with an increased risk of coronary heart disease [32]. Cross-sectional evidence from the Nurses' Health Study highlighted that *trans*-9 18:1 intake was positively associated with plasma biomarkers of inflammation and endothelial dysfunction, including E-selectin concentrations [33]. While we did not directly measure the HAEC membrane FA composition, it is plausible that the higher proportion of total *trans*-MUFA in the plasma lipid pool following intake of FA-modified dairy products in the human study may have counteracted some of the favourable changes observed in the postprandial FA pool with this treatment, including lower plasma 16:0 and higher *cis*-9 18:1 proportion. In mechanistic support of this, it was shown that exposure of human microvascular endothelial cells to physiologically relevant concentrations (≤ 0.1 mM) of elaidic acid, but not vaccenic acid, for 180 min was linked to increased NF-κB activation and impaired endothelial insulin signaling and NO production [34]. Furthermore, vaccenic acid had a neutral effect on E-selectin concentrations in both healthy and type 2 diabetic HAEC [7].

Strengths of the present study include the fact that we exposed HAEC to human plasma samples following longer-term ingestion of whole dairy foods that varied in FA composition; this helped to provide a more physiological assessment of mechanisms by which these treatments may affect CVD risk in vitro. Additionally, our study design took into consideration that the FA composition of the background diet may be of more relevance to cardiometabolic health than the isolated intake of dietary FA in acute meal settings [5]. However, our study presents some limitations. First, our in vivo study did not fully support the main human study finding of attenuated postprandial endothelial function following FA-modified dairy consumption. Second, as we did not employ an absolute quantification method for gene expression data [15], it was not possible to account for potential baseline (fasting) differences in target genes in response to the 12-week dairy exposures. This may partly explain inconsistencies between cell culture supernatant measures and gene expression data. Third, we did not directly measure the HAEC membrane FA composition following incubations and thus relied upon measures of total lipid FA proportion from our human plasma samples. Fourth, our study measured genes involved in lipid regulation and endothelial activation. However, measurement of gene expression does not provide insights into protein abundance or activity; these are strongly influenced by post-transcriptional events (including translation and protein degradation) that occur after mRNA is made [35]. Future work should consider using dynamic monitoring, rather than conventional static HAEC cell models, to allow for the recapitulation of physiological responses ex vivo.

## Conclusion

We found that incubation of HAEC with human plasma collected after the SFA-reduced, MUFA-enriched dairy treatment had a beneficial impact on NOx production, yet up-regulated relative gene expression of E-selectin. Further ex vivo research is needed to extend our knowledge on the effects of partial replacement of SFA with unsaturated FA in dairy foods on molecular alterations in the human endothelium, as these are of central importance in the pathophysiology of CVD.

### Supplementary Information

Below is the link to the electronic supplementary material.

Supplementary file1 (DOCX 370 kb)

## Data Availability

The data that support the findings of this study are available from the corresponding author upon reasonable request.

## Abbreviations

Apo: Apolipoprotein
BMI: Body mass index
CVD: Cardiovascular disease
eNOS: Endothelial nitric oxide synthase
FA: Fatty acid
FBS: Foetal bovine serum
FMD: Flow-mediated dilatation
HAEC: Human aortic endothelial cells
ICAM-1: Inter-cellular adhesion molecule-1
LDL-R: LDL-receptor
NEFA: Non-esterified fatty acids
NO: Nitric oxide
NOx: Total nitric oxide
OA: Oleic acid
PA: Palmitic acid
RESET: REplacement of SaturatEd fat in dairy on total cholesterol
SIRT1: Sirtuin 1
UHT: Ultra-high temperature
VCAM-1: Vascular cell adhesion molecule
%TE: Percentage of total energy intake
