# Supplementary material for: Impact of dairy fat manipulation on endothelial function and lipid regulation in human aortic endothelial cells exposed to human plasma samples: an in vitro investigation from the RESET study
Source: Eur J Nutr. 2023 Dec 13;63(2):539–48. doi: 10.1007/s00394-023-03284-9 (PMC10899290; doi:10.1007/s00394-023-03284-9)
Supplement: Supplementary file 1 — Supplementary file1 (DOCX 370 kb) [file 394_2023_3284_MOESM1_ESM.docx]

**Supplementary Table 1** Baseline (fasting) characteristics of the subset of participants selected for the *in vitro* cell study following each dietary intervention period^1^

| Characteristic | Modified | Control | |
| --- | --- | --- | --- |
|  | (*n* = 11) (*n* = 11) | | |
| Age, y | 57.5 ± 1.8 | | |
| Sex |  |  | |
| Men, *n* (%) | 6 (55) | | |
| Women, *n* (%) | 5 (45) | | |
| Ethnicity, *n* (%) |  | | |
| White | 11 (100) | | |
| CVD risk score^2^ | 3.3 ± 0.4 | | |
| Body mass, kg | 80.6 ± 3.9 | | 80.4 ± 3.9 |
| BMI, kg/m² | 26.1 ± 0.9 | 26.0 ± 0.9 | |
| Waist circumference, cm | 90.4 ± 3.9 | 93.2 ± 4.0 | |
| Blood pressure |  |  | |
| Systolic, mm Hg | 120 ± 4 | 119 ± 5 | |
| Diastolic, mm Hg | 70 ± 2 | 69 ± 3 | |
| Fasting circulating biomarkers |  |  | |
| TC, mmol/L | 5.74 ± 0.24 | 5.98 ± 0.25 | |
| LDL-C, mmol/L | 3.53 ± 0.19 | 3.67 ± 0.21 | |
| HDL-C, mmol/L | 1.66 ± 0.09 | 1.66 ± 0.09 | |
| TG, mmol/L | 1.22 ± 0.12 | 1.42 ± 0.16 | |
| ApoB, mg/mL | 1.00 ± 0.05 | 1.03 ± 0.07 | |
| NEFA, μmol/L | 557 ± 49 | 490 ± 43 | |
| Glucose, mmol/L | 5.29 ± 0.19 | 5.22 ± 0.15 | |
| Insulin, pmol/L | 35.1 ± 4.2 | 32.6 ± 4.2 | |
| Nitrite, μmol/L | 0.22 ± 0.06 | 0.08 ± 0.03 | |

^1^Values are given as unadjusted means ± SEMs or *n* (%). Ethnicity was determined by self-reporting. CVD, cardiovascular disease; LDL-C, LDL cholesterol; HDL-C, HDL cholesterol; TG, triacylglycerol; TC, total cholesterol. Paired t-tests, or non-parametric equivalent, were used to compare baseline (fasting) characteristics of participants following each 12-wk dietary intervention period with fatty acid-modified or conventional (control) dairy products.

^2^Assessed with the use of a modified Framingham risk score, where a score of ≥2 points relates to a 50% higher risk of CVD than the population mean [1].


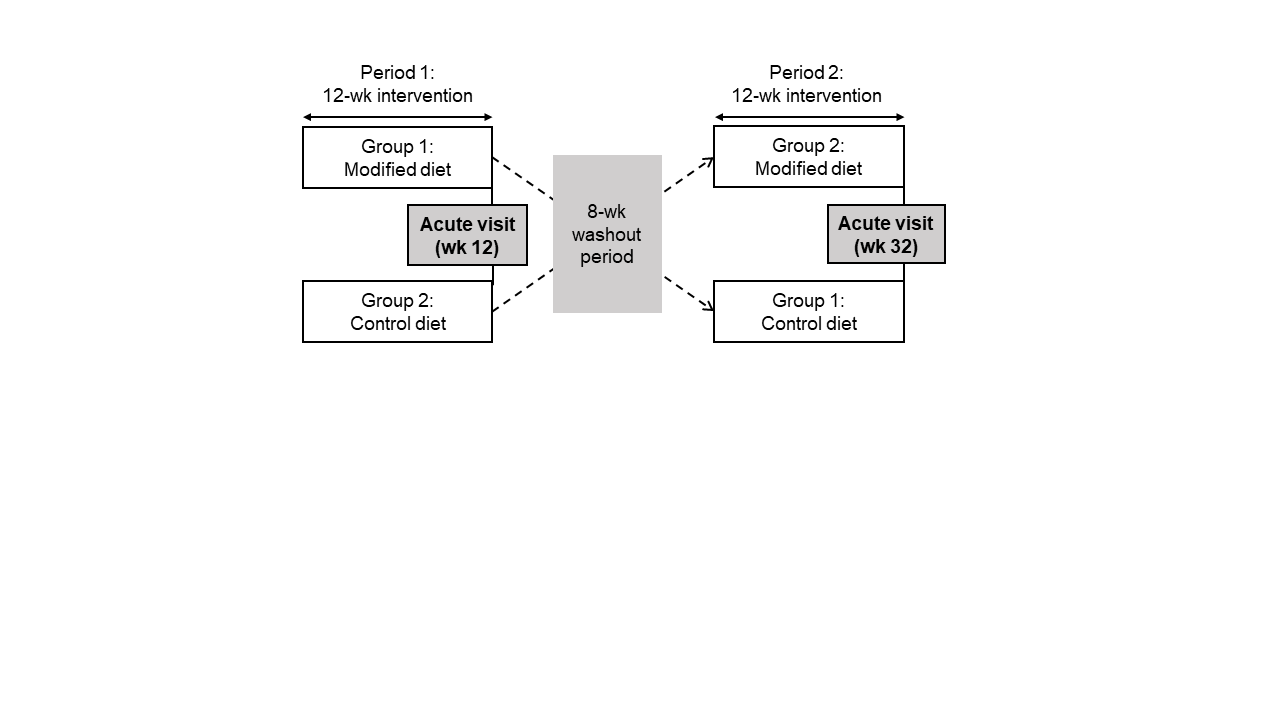


**Supplemental Fig. 1** Overview of study visits for the *in vitro* HAEC sub-study that was conducted within the framework of the RESET acute-within-chronic, crossover randomized controlled dietary trial. Participants were randomly assigned to group 1, where a postprandial visit (incorporating sequential meals rich in FA-modified dairy products) was completed after a 12-wk dietary intervention period with the same FA-modified dairy products (period 1), or group 2, where a postprandial visit [incorporating sequential meals rich in conventional (control) dairy products] was completed after a 12-wk dietary intervention period with the same control dairy products (period 1). Following an 8-wk washout period, participants crossed over to the alternate diet period and completed postprandial study visits before and after a 12-wk dietary intervention (period 2). FA, fatty acid; HAEC, human aortic endothelial cells; RESET, REplacement of SaturatEd fat in dairy on Total cholesterol


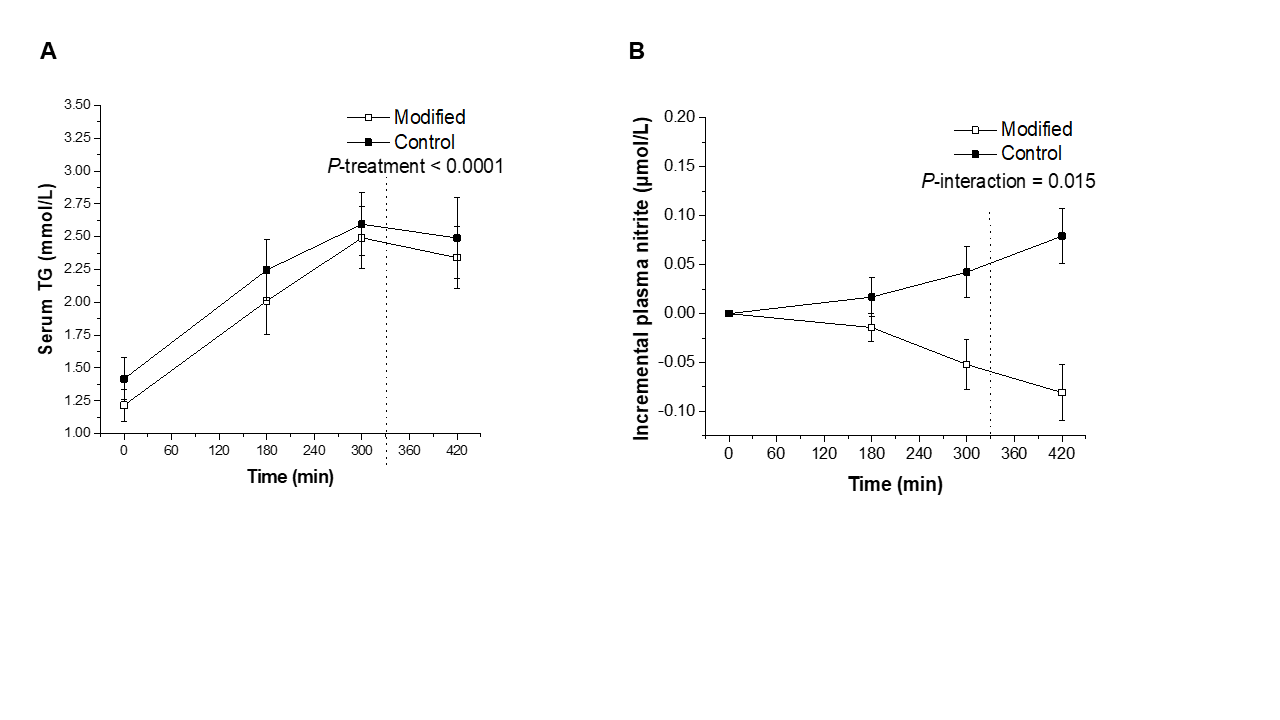


**Supplemental Fig. 2** Postprandial serum TG concentrations (A) and postprandial change in plasma nitrite concentrations (B) in response to sequential high-fat mixed-meal challenges (breakfast at 0 min and lunch at 330 min) representative of the FA-modified and conventional (control) dairy diets following each 12-wk dietary intervention in adults with moderate CVD risk. Values are untransformed and unadjusted means ± SEMs (*n* = 11; subset of RESET participants selected for the *in vitro* cell study). The dotted lines represent the timing of the second meal (330 min). Linear mixed-model analysis was used to explore the effects of treatment and time, with an adjustment made in all cases for fixed- (period, time, treatment, sex, age, and BMI) and random- (participant) effect covariates. For plasma nitrite, incremental postprandial treatment x time interactions/overall treatment effects were assessed by subtracting fasting (baseline) from postprandial values (i.e., the baseline value was treated as ‘0’) [2]. *P* < 0.05 was deemed significant. TG, triacylglycerol


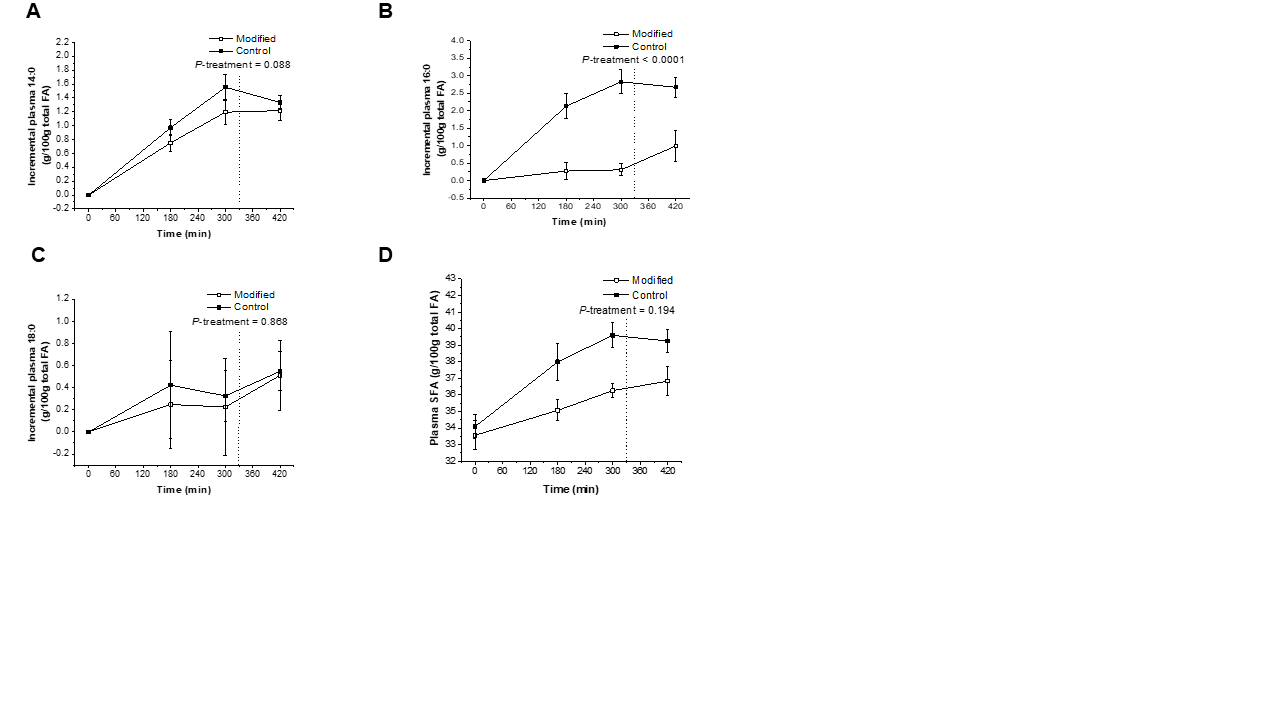


**Supplemental Fig. 3** Postprandial plasma total lipid 14:0 (A), 16:0 (B), 18:0 (C) and SFA (D) proportion in response to sequential high-fat mixed-meal challenges (breakfast at 0 min and lunch at 330 min) representative of the FA-modified and conventional (control) dairy diets following each 12-wk dietary intervention in adults with moderate CVD risk. Values are untransformed and unadjusted means ± SEMs (*n* = 11; subset of RESET participants selected for the *in vitro* cell study). The dotted lines represent the timing of the second meal (330 min). Linear mixed-model analysis was used to explore the effects of treatment and time, with an adjustment made in all cases for fixed- (period, time, treatment, sex, age, and BMI) and random- (participant) effect covariates. For plasma total lipid 14:0, 16:0, and 18:0, incremental postprandial treatment x time interactions/overall treatment effects were assessed by subtracting fasting (baseline) from postprandial values (i.e., the baseline value was treated as ‘0’) [2]. *P* < 0.05 was deemed significant. FA, fatty acid


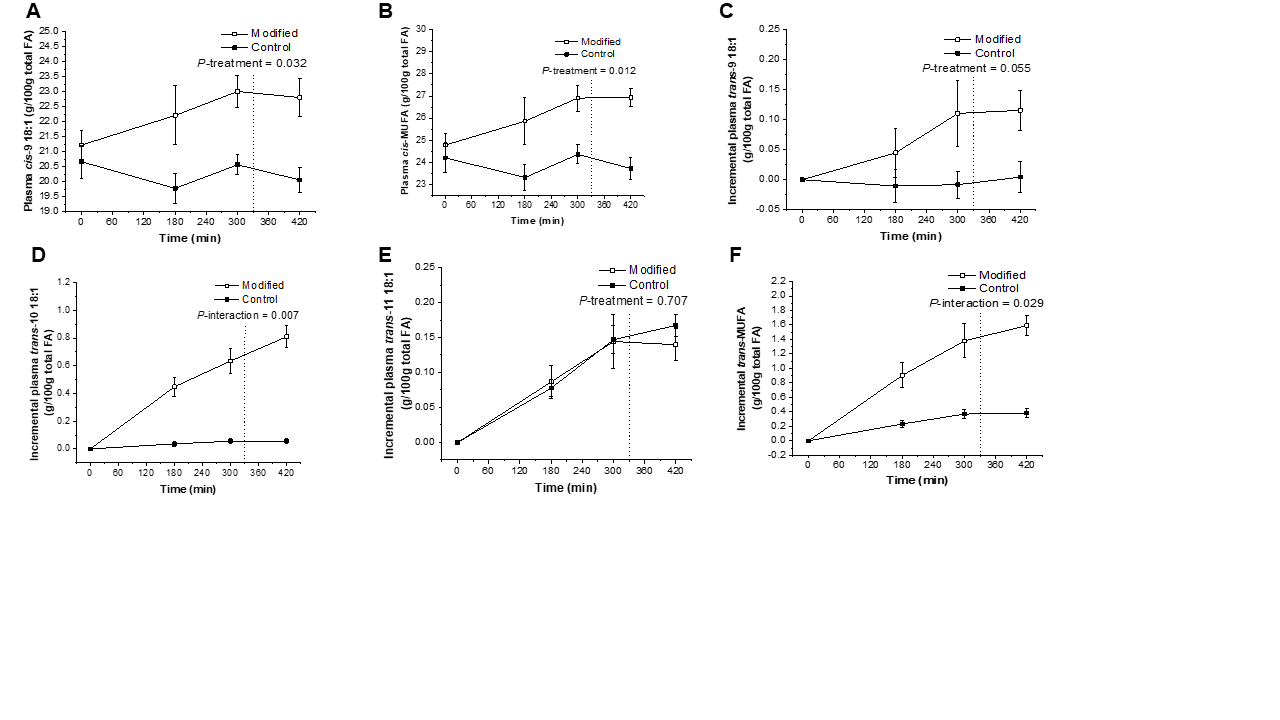


**Supplemental Fig. 4** Postprandial plasma total lipid *cis*-9 18:1 (A), *cis*-MUFA (B), *trans*-9 18:1 (C), *trans*-10 18:1 (D), *trans*-11 18:1 (E) and *trans*-MUFA (F) proportion in response to sequential high-fat mixed-meal challenges (breakfast at 0 min and lunch at 330 min) representative of the FA-modified and conventional (control) dairy diets following each 12-wk dietary intervention in adults with moderate CVD risk. Values are untransformed and unadjusted means ± SEMs (*n* = 11; subset of RESET participants selected for the *in vitro* cell study). The dotted lines represent the timing of the second meal (330 min). Linear mixed-model analysis was used to explore the effects of treatment and time, with an adjustment made in all cases for fixed- (period, time, treatment, sex, age, and BMI) and random- (participant) effect covariates. For plasma total lipid *trans*-9 18:1, *trans*-10 18:1, *trans*-11 18:1, and *trans*-MUFA, incremental postprandial treatment x time interactions/overall treatment effects were assessed by subtracting fasting (baseline) from postprandial values (i.e., the baseline value was treated as ‘0’) [2]. *P* < 0.05 was deemed significant. FA, fatty acid

**Reference**

1. Markey O, Vasilopoulou D, Kliem KE, Koulman A, Fagan CC, Summerhill K, Wang LY, Grandison AS, Humphries DJ, Todd S, Jackson KG, Givens DI, Lovegrove JA (2017) Plasma phospholipid fatty acid profile confirms compliance to a novel saturated fat-reduced, monounsaturated fat-enriched dairy product intervention in adults at moderate cardiovascular risk: a randomized controlled trial. Nutrition journal 16 (1):33. doi:10.1186/s12937-017-0249-2

2. Lairon D, Lopez-Miranda J, Williams C (2007) Methodology for studying postprandial lipid metabolism. Eur J Clin Nutr 61 (10):1145-1161. doi:10.1038/sj.ejcn.1602749
